# Vitamin C Supplementation Accelerates Healing in Diabetic Foot Ulcers: Insights From a Systematic Review of Clinical Evidence

**DOI:** 10.7759/cureus.109297

**Published:** 2026-05-20

**Authors:** Mohammed A Saad, Durgam Sathyam, Sravanthi Koora, Satyanarayana Kottireddy

**Affiliations:** 1 Department of Biochemistry, Government Medical College Karimnagar, Karimnagar, IND; 2 Department of Biochemistry, Government Medical College, Kumuram Bheem Asifabad, Asifabad, IND; 3 Department of Pharmacology, Surabhi Institute of Medical Sciences, Siddipet, IND

**Keywords:** ascorbic acid, diabetes mellitus, diabetic foot ulcer, micronutrient deficiency, vitamin c, wound healing

## Abstract

Diabetic foot ulcers (DFUs) persist as a predominant clinical challenge, constituting the primary etiological factor in the pathway to lower-extremity amputation and a significant source of comorbidity and mortality among individuals with diabetes mellitus. The role of vitamin C in wound repair is critical, yet its specific impact on DFUs requires further synthesis. Deficiencies in this micronutrient can disrupt collagen formation, immune response, and oxidative defense mechanisms, potentially impeding healing. To investigate this, a systematic review was conducted to assess clinical evidence linking vitamin C status, via supplementation or biochemical deficiency, to DFU prevalence and healing outcomes. A search strategy was executed in major electronic databases, identifying relevant clinical trials and observational studies up to September 2025. From the identified records, 10 studies were selected for final inclusion. The quality of evidence was subsequently evaluated using the Cochrane RoB 2.0 tool for randomized controlled trials (RCTs) and the Newcastle-Ottawa Scale for observational studies. Vitamin C supplementation (500-1000 mg/day) significantly enhanced DFU healing outcomes in all four RCTs, with improved wound closure, reduced ulcer size, and faster recovery compared with control groups. Observational data demonstrated that low serum vitamin C (<23 µmol/L) was strongly correlated with delayed healing, severe ulceration, and increased risk of amputation. Combination therapies using vitamin C with vitamin E, platelet-rich plasma (PRP), fibrin glue, or silver dressings yielded synergistic healing benefits. Vitamin C deficiency is highly prevalent among DFU patients and is consistently associated with poor healing outcomes. Oral supplementation, especially in deficient individuals, appears to enhance wound recovery. Future large-scale RCTs should determine optimal dosing and clarify mechanistic pathways for clinical translation.

## Introduction and background

The lifetime incidence of diabetic foot ulcers (DFUs) had previously been estimated at 15-25% among adults with diabetes; however, more recent and extensive investigations imply that the risk may be higher, with 19-34% of patients likely to develop a foot ulcer during their lifetime [1]. This heterogeneity is due to variances in research demographics, healthcare access, and regional disease burden, as well as distinctions between Type 1 and Type 2 diabetes. DFUs continue to be a major cause of morbidity, frequently resulting in prolonged hospitalization, recurrent infection, and non-traumatic lower-limb amputation, all of which incur significant healthcare costs and decrease quality of life [1]. Historically, DFU etiology has been dominated by peripheral neuropathy, repetitive mechanical stress, foot deformity, and peripheral arterial disease; these pathophysiologic factors combine with impaired immune responses to produce chronic, nonhealing wounds [1]. The global burden of diabetes mellitus has seen a substantial increase over the past decade, thereby amplifying the prevalence and impact of its associated complications, and consequently, the number of patients at risk of DFU and its complications has increased; global diabetes prevalence rose substantially between earlier International Diabetes Federation (IDF) reports and the most recent Atlas data [2].

The management of DFUs has progressively evolved beyond traditional measures of debridement, off-loading, and infection control. Modern protocols now emphasize standardized risk stratification, early revascularization, and a multidisciplinary team approach, supplemented by advanced technologies like negative-pressure wound therapy, bioactive dressings, and cellular therapies [3,4]. These advances have improved limb-salvage rates in specialist centers, but overall healing outcomes remain suboptimal for many patients, particularly those with multimorbidity, malnutrition, or unrecognized micronutrient deficiencies [3,5].

Nutrition and micronutrient sufficiency are increasingly recognized as modifiable determinants of wound healing. Ascorbic acid (vitamin C) serves as an essential cofactor for the enzymes prolyl and lysyl hydroxylase, which catalyze the hydroxylation of proline and lysine residues in procollagen [6,7]. Beyond this structural role, vitamin C exerts antioxidant activity, enhances immune cell function, and supports angiogenesis -- processes that are impaired in chronic diabetic wounds [6,7]. Observational cohorts of DFU patients report a high prevalence of micronutrient insufficiency, and low serum ascorbate concentrations are correlated with greater ulcer severity, delayed healing, and elevated risks of infection and limb amputation [8,9]. Clinical intervention studies have explored whether replenishing or supplementing vitamin C can enhance DFU healing. Randomized trials and pilot studies have reported faster ulcer area reduction and higher rates of closure with oral vitamin C (usually 500 mg/day), and combination strategies (vitamin C plus vitamin E, or vitamin C with platelet-rich plasma (PRP)/fibrin glue or advanced dressings) have shown additive benefits in some trials [10-13]. Nonetheless, studies differ in design, dosage, population characteristics, and concurrent wound care, and many are small or single-center, leaving uncertainty about the magnitude and generalizability of effect.

Nonetheless, available studies vary greatly in design, sample size, patient characteristics, vitamin C dose regimens, duration of intervention, and outcome measures, which restricts the capacity to draw consistent conclusions and generalize findings. This variation underlines the necessity for a rigorous synthesis of the current clinical evidence. This review gathers clinical evidence from randomized controlled trials (RCTs), cohort studies, cross-sectional analyses, and case-control investigations that studied vitamin C supplementation or serum vitamin C levels in individuals with DFUs. By synthesizing data from rigorously designed clinical studies, the review aims to determine the prevalence of vitamin C deficiency among individuals with DFUs, to assess the relationship between serum ascorbate concentration and healing outcomes, and to evaluate whether oral vitamin C supplementation, either alone or in combination with other therapeutic approaches, improves clinically significant outcomes such as time to wound closure, percentage reduction in ulcer area, infection occurrence, and amputation rate. The findings are expected to help clinicians understand the role of nutritional assessment and targeted micronutrient therapy in comprehensive DFU management and to highlight the need for future large-scale clinical investigations.

## Review

Methodology

Protocol

**Figure 1:**
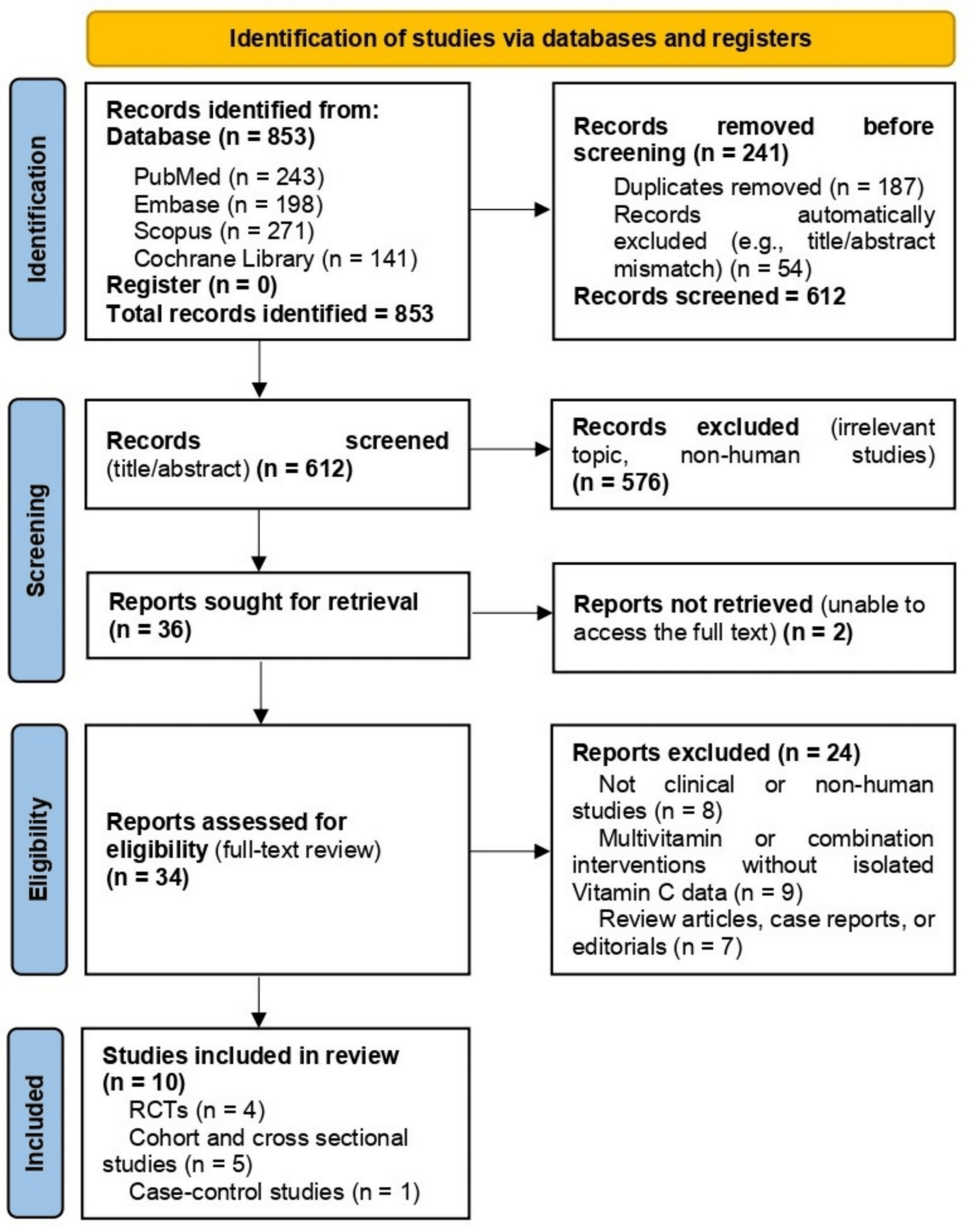
PRISMA flow diagram for literature search and article inclusion. The PRISMA flow diagram summarizes the study selection process. A total of 853 records were identified from four databases. After removing duplicates and screening titles and abstracts, 34 full-text articles were assessed for eligibility. Finally, 10 studies met the inclusion criteria and were included in this systematic review. PRISMA, Preferred Reporting Items for Systematic Reviews and Meta-Analyses.

This systematic review followed the recommendations outlined in the Preferred Reporting Items for Systematic Reviews and Meta-Analyses (PRISMA) to maintain methodological rigor, transparency, and reproducibility [14]. A predefined protocol was carefully prepared and examined by all members of the review team to ensure consistency and uniformity during each stage of the review process. The protocol outlined the study objectives, specified the inclusion and exclusion parameters, detailed the database search approach, described the data extraction procedure, and defined the methods used for quality assessment. Although the protocol was not formally registered in PROSPERO, the review strictly adhered to evidence-based procedures typically required for registered systematic reviews (Figure 1).

Eligibility Criteria

Studies were deemed eligible for inclusion if they involved adult participants aged 18 years or older with a confirmed diagnosis of diabetes mellitus and at least one DFU. Eligible study designs included RCTs, cohort investigations, cross-sectional analyses, and case-control studies that explored the effect of vitamin C supplementation or assessed serum vitamin C levels in relation to wound-healing outcomes. The primary outcomes of interest were ulcer healing rate, time to closure, percentage reduction in ulcer area, and amputation rate. Secondary outcomes included oxidative stress markers, inflammatory parameters, and infection rates. Only articles published in English between January 2000 and September 2025 were included. Studies were excluded if they involved animal or laboratory experiments, review papers, conference abstracts, or editorials. Research focused on non-diabetic wounds or multivitamin interventions that did not separately evaluate the effect of vitamin C was also omitted.

Search Strategy

An extensive literature search was conducted using four major electronic databases: PubMed, Embase, Scopus, and the Cochrane Library. The search strategy employed a combination of controlled vocabulary terms and free-text keywords, structured with Boolean operators to enhance precision. The key search phrases included “Vitamin C,” “ascorbic acid,” and “antioxidant vitamin,” which were paired with terms such as “diabetic foot ulcer,” “DFU,” and “diabetic wound,” along with outcome-related keywords including “healing,” “wound closure,” “clinical trial,” and “observational study. The strategy was tailored to suit each database. Furthermore, the reference lists of all included papers were examined manually to locate any additional studies that met the eligibility criteria. The comprehensive database search yielded 853 records in total, with 243 from PubMed, 198 from Embase, 271 from Scopus, and 141 from the Cochrane Library. After duplicate removal and preliminary screening of titles and abstracts, 34 articles were retained for full-text assessment. Of these, 10 studies satisfied all inclusion requirements and were incorporated into the final review.

Data Extraction and Study Selection

Two independent reviewers evaluated all titles and abstracts to determine relevance and subsequently examined the full texts of studies that appeared to meet the inclusion criteria. Data extraction was also conducted independently using a predesigned standardized template to ensure reliability and uniformity. The extracted information encompassed details such as author names, year of publication, study design, sample size, participant characteristics, type of intervention or exposure, vitamin C dosage or serum concentration, treatment duration, ulcer classification, main and secondary outcomes, and summarized results. Any disagreements between reviewers were resolved through discussion, and when necessary, by consulting a third reviewer to uphold objectivity and reduce potential bias.

Bias Assessment Risk

The quality of the included studies and the potential risk of bias were evaluated using validated assessment tools tailored to each study design. For RCTs, the Cochrane Risk of Bias 2.0 tool was utilized to assess key domains, including the randomization process, deviations from intended interventions, completeness of outcome data, accuracy of outcome measurement, and selective reporting [15]. Each domain was categorized as presenting a low, moderate, or high risk of bias. For observational studies, methodological rigor was examined using the Newcastle-Ottawa Scale (NOS), which evaluates three core areas: participant selection, comparability of study groups, and determination of exposure or outcome [16]. Studies with a total NOS score of seven or above were regarded as high quality.

In general, the RCTs included in this review demonstrated a low overall risk of bias, although some exhibited limitations related to small sample sizes and partial blinding. Most observational studies were rated as moderate to high in quality, with common concerns arising from potential confounding variables such as variations in glycemic control, nutritional status, and coexisting comorbidities. The complete study selection process, along with reasons for exclusion, is illustrated in the PRISMA flow diagram, which presents the number of records identified, screened, excluded, and ultimately included in the review.

Data synthesis: A formal quantitative meta-analysis was not performed due to significant clinical and methodological heterogeneity among the included studies, particularly in terms of intervention type (vitamin C monotherapy versus combination therapies), dosage (500-1000 mg/day), treatment duration, outcome measures (ulcer size reduction, complete healing, time to closure), and study designs (RCTs and observational studies). Instead, a narrative synthesis approach was used to comprehensively synthesize and analyze the findings from many investigations. This strategy was deemed appropriate due to the heterogeneity in research characteristics and inconsistent reporting of effect sizes and outcome measures, which hampered the feasibility of statistical pooling. 

Results

Study Selection and Characteristics

A total of 10 clinical studies met the inclusion criteria for this systematic review, consisting of four RCTs [10-13], five cohort [8,9,17,18] or cross-sectional investigations [19], and one case-control study [20]. Sample sizes ranged from 13 to 212 participants, with intervention durations between six and 12 weeks. The studies were conducted across diverse clinical settings and geographic regions, including Australia, India, Iran, Turkey, and the United States. Most trials evaluated 500-1000 mg/day of oral vitamin C supplementation, either as monotherapy or in combination with vitamin E or advanced wound dressings, while the remaining studies assessed serum vitamin C concentrations and their relationship with ulcer-healing outcomes [8,11].

Across designs, there was substantial consistency in demonstrating that vitamin C sufficiency was associated with improved wound healing, reduced ulcer area, and shorter healing duration, while deficiency correlated with greater ulcer severity, delayed recovery, and higher amputation risk [9,17].

Findings From RCTs

Four RCTs investigated the therapeutic role of vitamin C supplementation in the management of DFU. In a key study by Gunton et al. [11], participants who received 500 mg of oral vitamin C daily achieved complete ulcer closure in all cases, whereas only 56% of patients in the placebo group reached full healing within eight weeks, indicating a notable improvement in tissue repair. In another trial, Vasanthi and Goldlin [10] reported a 72.5% reduction in ulcer area among individuals treated with a combination of vitamin C (1000 mg per day) and vitamin E (800 IU per day), compared with 39.7% in the control group, highlighting a synergistic antioxidant effect that promoted wound recovery. Similarly, Yarahmadi et al. [12] combined vitamin C and vitamin E supplementation with PRP and fibrin glue therapy, demonstrating enhanced epithelialization and oxidative balance in patients with chronic, non-healing ulcers. This combined therapy significantly accelerated epithelialization, improved oxidative stress profiles, and reduced wound area, suggesting that antioxidants can potentiate biologic wound therapies.

Likewise, Lafontaine et al. [13] demonstrated that patients receiving silver dressings and oral vitamin C supplementation exhibited faster ulcer closure and improved granulation tissue formation compared with those managed by standard care alone. Collectively, these RCTs indicate that vitamin C supplementation, either alone or as part of a multimodal approach, can meaningfully enhance DFU healing outcomes.

Findings From Cohort and Cross-Sectional Studies

Five observational studies explored how serum vitamin C concentrations correlate with ulcer severity and healing outcomes. In a prospective cohort involving 131 individuals with DFU, Pena et al. [8] observed that participants with serum vitamin C levels lower than 23 µmol/L tended to present with more advanced ulcer grades and exhibited slower rates of wound recovery compared with those who had sufficient levels. Similarly, Brookes et al. [9] found that low serum vitamin C was an independent predictor of lower-limb amputation, even after adjusting for age, glycemic control, and vascular status. In a multicenter investigation, Erdem et al. [17] found that approximately 64% of patients with DFU exhibited vitamin C deficiency, which was significantly linked to slower wound healing and an increased likelihood of amputation.

Tong et al. [19] explored correlations between vitamin C, glycemic control, and skin microbiome diversity, finding that lower serum ascorbate levels were linked to elevated HbA1c and dysbiosis in chronic ulcers. In a separate prospective analysis, Kancherla et al. [18] reported that administering vitamin C supplementation at a dose of 500 mg per day for six weeks before surgery enhanced postoperative wound healing and lowered infection incidence. Collectively, the evidence indicates that vitamin C deficiency is widespread among individuals with DFU and that restoring or maintaining adequate vitamin C levels may positively impact wound repair and overall healing outcomes.

Findings From a Case-Control Study

In a case-control study, Bolajoko et al. [20] compared 70 individuals with DFU to 50 healthy participants and found that the mean serum vitamin C concentration was markedly lower in the DFU group (3.8 µmol/L) compared with controls (5.6 µmol/L, corresponding to a 0.3% probability level), indicating a significant association between vitamin C deficiency and ulcer occurrence. This result reinforces the notion that chronic diabetic wounds are frequently accompanied by suboptimal antioxidant status, which may contribute to impaired collagen synthesis, delayed angiogenesis, and persistent inflammation [6].

Comparative Synthesis of Findings

Across the 10 studies (Table 1), vitamin C supplementation (500-1000 mg/day) consistently improved ulcer healing rates, reduced wound size, and accelerated closure times compared with controls. Observational studies revealed that vitamin C deficiency was independently associated with poor healing, severe ulcer grades, and increased risk of amputation. The magnitude of benefit appeared greater among patients with baseline hypovitaminosis C, suggesting that targeted supplementation may be most effective in deficient populations.

**Table 1:** Summary of clinical evidence on the impact of vitamin C in the management of DFU This table provides a detailed overview of 10 clinical studies that investigated the relationship between vitamin C and DFU healing. It includes information on study design, participant characteristics, vitamin C dosage or serum concentrations, treatment duration, and primary clinical outcomes. The summarized findings highlight the therapeutic relevance of vitamin C supplementation and its association with improved wound healing parameters across diverse study populations. RCTs, randomized controlled trials; PRP, platelet-rich plasma; DFU, diabetic foot ulcer.

Study (Year)	Design/Sample (n), Duration	Intervention/Measure	Dose/Serum Level	Outcomes & Key Findings
Vasanthi & Goldlin, 2018 [10]	RCT; n=60; 12 weeks	Vitamin C + E vs control	E 400 IU ×2/day; C 500 mg ×2/day	72.5% vs 39.7% ulcer area reduction
Gunton et al., 2021 [11]	RCT; n=16; 8 weeks	Oral vitamin C vs placebo	500 mg/day	100% healing in vitamin C group vs 56% in placebo
Yarahmadi et al., 2021 [12]	RCT; n=13; 8 weeks	PRP + fibrin glue + Vit E + C	E 400 IU/day; C 500 mg/day; PRP 3 mL; fibrin glue topical	Accelerated ulcer healing and improved oxidative markers
Lafontaine et al., 2023 [13]	RCT; n=70; 8 weeks	Silver dressing + Vit C vs standard care	500 mg/day	Faster ulcer closure compared to standard care
Peña et al., 2020 [8]	Cohort; n=131	Serum vitamin C levels	Deficient <23 µmol/L	Low Vit C associated with severe DFUs
Brookes et al., 2020 [9]	Cohort; n=82	Serum vitamin C levels	Deficient <23 µmol/L	Deficiency predicted amputation risk
Erdem et al., 2024 [17]	Cohort; n=212	Serum vitamin C levels	64% deficient	Linked to delayed healing and increased amputation
Kancherla et al., 2025 [18]	Cohort; n=61; 6 weeks	Pre-op oral vitamin C	500 mg/day	Higher Vit C linked with faster healing and fewer infections
Tong et al., 2022 [19]	Cross-sectional; n=75	Serum vitamin C levels	Mean 20 µmol/L	Low Vit C correlated with poor HbA1c and altered microbiome
Bolajoko et al., 2017 [20]	Case-control; n=70 DFU vs 50 controls	Serum vitamin C	DFU: 3.8 vs controls: 5.6 µmol/L	DFU patients had significantly lower Vit C levels

Discussion

This systematic review consolidates high-quality clinical data, establishing vitamin C as a key determinant in both preventing the onset and improving the outcomes of DFUs. Across 10 clinical studies, both interventional and observational designs consistently demonstrated that sufficient vitamin C levels accelerate wound healing, while deficiency correlates strongly with delayed repair, ulcer recurrence, and a higher risk of amputation. The consistency of these findings across study types and populations reinforces the biological plausibility of vitamin C’s role as a critical determinant of wound-healing outcomes in diabetes.

Vitamin C is a vital cofactor for the enzymes that hydroxylate proline and lysine, a requisite step for the assembly of stable collagen fibrils and the subsequent development of tensile strength in granulation tissue [6,7]. In addition, it supports fibroblast proliferation, promotes angiogenesis, enhances neutrophil function, and acts as a potent antioxidant by neutralizing reactive oxygen species (ROS). In diabetic patients, persistent hyperglycemia increases oxidative stress, leading to the depletion of endogenous vitamin C and impaired endothelial repair mechanisms [21]. This biochemical rationale aligns with clinical findings showing improved ulcer outcomes following vitamin C supplementation.

The RCTs included in this review consistently reported favorable outcomes for vitamin C supplementation. Notably, Gunton et al. [11] observed a significantly higher rate of complete wound closure in the intervention group receiving oral vitamin C compared to the placebo group (100% vs. 56%), a finding that suggests a potential dose-dependent relationship with wound resolution. Similarly, Lafontaine et al. [13] showed that the addition of 500 mg/day of vitamin C to silver dressings accelerated ulcer closure and improved granulation tissue formation, supporting the adjunctive use of micronutrients with advanced wound dressings. Vasanthi and Goldlin [10] found that combined supplementation of vitamins C and E significantly reduced ulcer area by 72.5%, compared with 39.7% in controls, highlighting synergistic antioxidant effects. The study by Yarahmadi et al. [12] also reported that co-administration of vitamins C and E with PRP and fibrin glue enhanced epithelialization and oxidative balance, underscoring the value of combination therapy in complex wound healing.

Observational studies reinforced the therapeutic significance of maintaining adequate vitamin C status. Pena et al. [8] reported that patients with serum vitamin C levels below 23 µmol/L experienced slower ulcer healing and greater lesion severity. Brookes et al. [9] identified low vitamin C levels as an independent predictor of amputation, even after adjusting for glycemic and vascular parameters. Erdem et al. [17] found that 64% of DFU patients were deficient in vitamin C, linking hypovitaminosis C with delayed epithelial repair and increased amputation rates. Likewise, Tong et al. [19] observed that lower ascorbate concentrations were associated with poor glycemic control (higher HbA1c) and reduced skin microbiome diversity, suggesting a systemic metabolic influence on wound pathology. Collectively, these findings emphasize that assessing vitamin C status should be an integral component of DFU evaluation and management.

The underlying biological mechanisms are well recognized, as vitamin C facilitates angiogenesis through stabilization of hypoxia-inducible factor 1α (HIF-1α) and upregulation of vascular endothelial growth factor (VEGF) [22]. It also improves immune defense by stimulating leukocyte migration and phagocytosis while reducing pro-inflammatory cytokine levels such as IL-6 and TNF-α [23]. These actions are particularly relevant in DFUs, where chronic inflammation, microvascular dysfunction, and impaired collagen cross-linking collectively hinder healing. Therefore, correcting vitamin C deficiency can simultaneously address oxidative stress, inflammation, and extracellular matrix repair -- the three central barriers to wound closure in diabetes.

Another critical insight from this review is the high prevalence of vitamin C deficiency among diabetic populations. Multiple cohort studies demonstrated that hypovitaminosis C is widespread in DFU patients, with prevalence rates ranging from 50% to 70% [8,17]. These findings are consistent with broader epidemiological data showing reduced circulating ascorbate in individuals with type 2 diabetes due to increased oxidative consumption and reduced intestinal absorption [24]. This suggests that even moderate supplementation (500-1000 mg/day) may restore plasma levels sufficient to promote wound repair and enhance microvascular function.

The clinical implications of these findings are substantial. DFU remains a leading cause of lower-limb amputation, accounting for 85% of diabetes-related amputations globally [1]. Despite advances in wound care and infection control, healing rates remain suboptimal, and recurrence is common. The integration of vitamin C assessment and supplementation into DFU treatment protocols offers a low-cost, safe, and potentially effective adjunctive strategy. Such an approach aligns with precision nutrition principles and could reduce both healing time and healthcare burden. Furthermore, perioperative vitamin C supplementation, as reported by Kancherla et al. [18], may enhance surgical wound recovery and reduce postoperative infections, extending its relevance beyond chronic ulcers.

This review has several limitations. Many included studies had small sample sizes, limiting generalizability. Considerable heterogeneity in supplementation dose, treatment duration, baseline vitamin C status, and outcome measures made direct comparison and quantitative synthesis difficult. Additionally, confounding factors such as diet, smoking, and concurrent medications were not consistently controlled, particularly in observational studies.

A further limitation is the absence of a quantitative meta-analysis. Despite the inclusion of multiple RCTs, substantial clinical and methodological variability, along with inconsistent reporting of effect sizes and confidence intervals, precluded reliable statistical pooling or meta-regression. Therefore, a narrative synthesis approach was adopted. Future large-scale studies with standardized methodologies are needed to enable meta-analysis and provide more precise estimates of treatment effects.

## Conclusions

In conclusion, this systematic review reinforces the clinical and biological significance of vitamin C in diabetic wound healing. Adequate vitamin C status supports collagen formation, enhances immune response, mitigates oxidative stress, and accelerates tissue regeneration. Deficiency, on the other hand, is a modifiable risk factor for poor healing and amputation. Routine screening for serum vitamin C and early supplementation could therefore be incorporated into standard DFU care algorithms, offering a practical, evidence-based means to improve patient outcomes.
